# Bibliometric mapping of non-invasive brain stimulation techniques (NIBS) for fluent speech production

**DOI:** 10.3389/fnhum.2023.1164890

**Published:** 2023-06-22

**Authors:** Wesley Medeiros, Tayná Barros, Fabio V. Caixeta

**Affiliations:** Laboratory of Neuroscience and Behavior, Department of Physiological Sciences, Institute of Biological Sciences, University of Brasilia, Brasília, Brazil

**Keywords:** neuromodulation, speech therapy, motor rehabilitation, scientometrics, stutter

## Abstract

**Introduction:**

Language production is a finely regulated process, with many aspects which still elude comprehension. From a motor perspective, speech involves over a hundred different muscles functioning in coordination. As science and technology evolve, new approaches are used to study speech production and treat its disorders, and there is growing interest in the use of non-invasive modulation by means of transcranial magnetic stimulation (TMS) and transcranial direct current stimulation (tDCS).

**Methods:**

Here we analyzed data obtained from Scopus (Elsevier) using VOSViewer to provide an overview of bibliographic mapping of citation, co-occurrence of keywords, co-citation and bibliographic coupling of non-invasive brain stimulation (NIBS) use in speech research.

**Results:**

In total, 253 documents were found, being 55% from only three countries (USA, Germany and Italy), with emerging economies such as Brazil and China becoming relevant in this topic recently. Most documents were published in this last decade, with 2022 being the most productive yet, showing brain stimulation has untapped potential for the speech research field.

**Discussion:**

Keyword analysis indicates a move away from basic research on the motor control in healthy speech, toward clinical applications such as stuttering and aphasia treatment. We also observe a recent trend in cerebellar modulation for clinical treatment. Finally, we discuss how NIBS have established over the years and gained prominence as tools in speech therapy and research, and highlight potential methodological possibilities for future research.

## Introduction

The wealth of readily available information is one of the hallmarks of our age. As any research field grows, growth becomes an important measure for science (Moral-Muñoz et al., 2020), allowing scientists to identify new trends in research, hotspots of production, and collaboration clusters around the world. Bibliometric (or scientometric) mapping is a systematic and unbiased way to visually represent the structure and dynamics of a specific research field based on citations, co-citations, and keywords, which are based on the quantitative analysis of published data. Bibliographic coupling identifies similarities in scientific articles based on their interlinked references. By analyzing shared references from multiple articles, it is possible to determine how closely these articles are related to each other and how they contribute to a given field of research. By examining the bibliographic coupling between a set of documents, it is possible to identify groups of related research and follow the evolution of a given field over time (van Eck and Waltman, 2017; José de Oliveira et al., 2019). Examples of software that perform this type of analysis are CiteSpace, bibExcel, and VOSViewer (Sun et al., 2022a).

Fluent speech refers to the production of speech that is smooth, effortless, and coherent, characterized by the absence of speech disruptions such as hesitations, repetitions, revisions, and dysfluencies. Its production relies on the seamless coordination of cognitive, linguistic, and motor control of multiple structures including the diaphragm, larynx, tongue, and lips (Neef et al., 2015). Relevant brain regions involved in this process are the left inferior frontal gyrus (LIFG, historically known as Broca's area) and the premotor cortices, which implement speech motor plans required to convey spoken language; and the posterior superior temporal gyrus, responsible for storing phonetic representations (or sound “blueprints”), is involved in auditory learning (Hickok and Poeppel, 2007; Hickok, 2012). This neural pathway, known as the “dorsal stream”, is left-lateralized in the brain of most people (Hickok and Poeppel, 2007; Hickok, 2012) and is central in the study of the neurobiology of speech production.

Non-invasive brain stimulation (NIBS) techniques are divided into two main groups as follows: transcranial magnetic stimulation (TMS), which employs magnetic fields to modulate the brain excitability, and transcranial electrical stimulation (TES), which applies electrical currents directly to the scalp in order to achieve neural modulation (Polanía et al., 2018). TES techniques include transcranial direct current stimulation (tDCS), transcranial alternating current stimulation (tACS), and transcranial random noise stimulation (tRNS). NIBS was first developed in the context of motor control research, and the seminal research by Pascual-Leone and collaborators in the 90s, showing the therapeutic effect of repeated TMS (rTMS) on major depression (Pascual-Leone et al., 1996), sparked an upsurge of interest in NIBS, both in clinical and experimental settings.

NIBS techniques can be applied to investigate causality between brain areas and specific processes (such as in speech), given that they modify brain activity in a temporary and reversible manner. This allows researchers to promote or inhibit local neuronal activation, to effectively simulate cortical lesions and modulate brain activity painlessly (Nitsche and Paulus, 2000, 2001; Woods et al., 2016; Fertonani and Miniussi, 2017). The sheer increase in publications observed in the last decade makes it clear that NIBS techniques have been established as valuable tools to study language (Flöel et al., 2008; Nitsche et al., 2008; Fiori et al., 2011; Jacobson et al., 2012; Monti et al., 2013; Rufener et al., 2017, 2019; Zoefel and Davis, 2017; Balboa-Bandeira et al., 2021; Sun et al., 2022b) and language disorders such as aphasia (Torres et al., 2013; Turkeltaub, 2015; Meinzer et al., 2016) and stuttering (Thiel et al., 2006; Chesters et al., 2018; Busan et al., 2019; Karsan et al., 2022).

Here, we provide a bibliometric coupling analysis of the studies on the use of NIBS for the motor aspects of speech since its beginning in the 90s. Although such analysis does not answer any specific research question, it may contribute to advances in the field by (1) helping researchers and stakeholders to understand the structure, development, and interconnections of scientific knowledge; (2) uncovering patterns of collaboration and novel potential intersections, fostering interdisciplinary research and innovation; (3) informing research policy, resource allocation, and strategic decision-making at the institutional, national, and international levels based on research impact; and (4) highlighting research gaps and guide future research directions as well as novel topics worth exploring in further studies (José de Oliveira et al., 2019; Donthu et al., 2021).

## Materials and methods

Data obtained from Elsevier (Scopus) were collected and used for a global analysis of the literature on NIBS and speech fluency. The search parameters used in the ‘*Advanced document search'* in Scopus were: TITLE-ABS-KEY (transcranial AND electrical OR magnetic OR current AND stimulation AND motor) AND TITLE-ABS-KEY(speech AND NOT perception AND NOT “deep brain stimulation”). The access date was April 2023. Additional parameters used in VOSViewer for the analysis of the data extracted from Scopus are presented in Table 1.

**Table 1:** Parameters used in VOSViewer for analysis of bibliography gathered from Scopus.

		**#**	**Minimum documents**	**Number meeting threshold**	**With links**	**Clusters**
1994–2012	Countries	26	2	12	10	3
	Keywords	153	2	26	21	6
	Citation–documents	72	5	62	28	7
2013–2023	Countries	38	3	20	19	5
	Keywords	469	5	31	22	5
	Citation–documents	185	7	106	63	10

Initially, a total of 363 documents were acquired in Scopus using the search parameters indicated above. Although the search string focused on NIBS and motor control of speech, some uncorrelated studies contained our keywords in the title or abstract. We individually examined each document to assess its thematic coherence with our research, specifically focusing on articles that explored the use of NIBS applied to treat or enhance speech production, including review articles. Studies that did not include NIBS and speech production in the experimental design (or review scope) were removed (110 removals). A final subset of 253 documents was selected for further analysis. Due to a noticeable change in the scope and methods employed in most studies in the 2010s (discussed further in the Results section), data were further divided into two-time segments, spanning from 1994 to 2011 and from 2012 to 2023.

In our analyses, we used VOSviewer (version 1.6.18), a software tool designed for mapping scientific landscapes: bibliometric maps can visually represent the network interactions of a field, illustrating the relationships between different areas, subfields, and research clusters, by means of plotting keyword co-occurrence and citation pattern. Co-occurrence refers to the presence of two or more keywords in the same document. Information of interest comprised the year of publication, language, country, title, author, affiliation, keywords, document type, abstract, and citations. All 253 results were exported in CSV format and synthesized into visual displays in VOSViewer.

For a network view, each item is represented by a circle. The size of an item's circle is determined by its influence. The color of an item is determined by the group of items belonging to the lines between the items that represent the correlation. In general, the closer two items are located, the stronger their relationship. The strongest co-citation links between items are also represented by the lines.

## Results

We found a marked difference in our analysis regarding the scope of studies across time. Particularly, from 1994 to 2011, the focus of research on NIBS techniques was mainly the use of transcranial magnetic stimulation (TMS) to address neural mechanisms underlying speech production and perception in healthy subjects. For instance, the first published paper that met our search criteria was *Speech localization using repetitive transcranial magnetic stimulation* (Jennum et al., 1994). During this period, researchers also began to explore tDCS in speech research, demonstrating that the technique could modulate cortical excitability and improve speech perception and production (Pulvermüller, 2005; Flöel et al., 2008).

From 2012 to 2023, there has been an increased focus on the use of NIBS techniques for the treatment of speech and language disorders. In particular, there has been growing interest in the use of tDCS with clinical applications for post-stroke speech rehabilitation, stuttering, and aphasia and also neurodegenerative diseases such as Parkinson's and other diseases that entail language loss (Holland and Crinion, 2012; Brewer et al., 2013; Flöel, 2014). Overall, the evolution of the first decade of NIBS was characterized by the use of TMS and its refinement and by the emergence of tDCS for speech research. In the following decade, the focus shifted to the development of protocols that concentrated on clinical applications for speech and language disorders.

**Figure 1:**
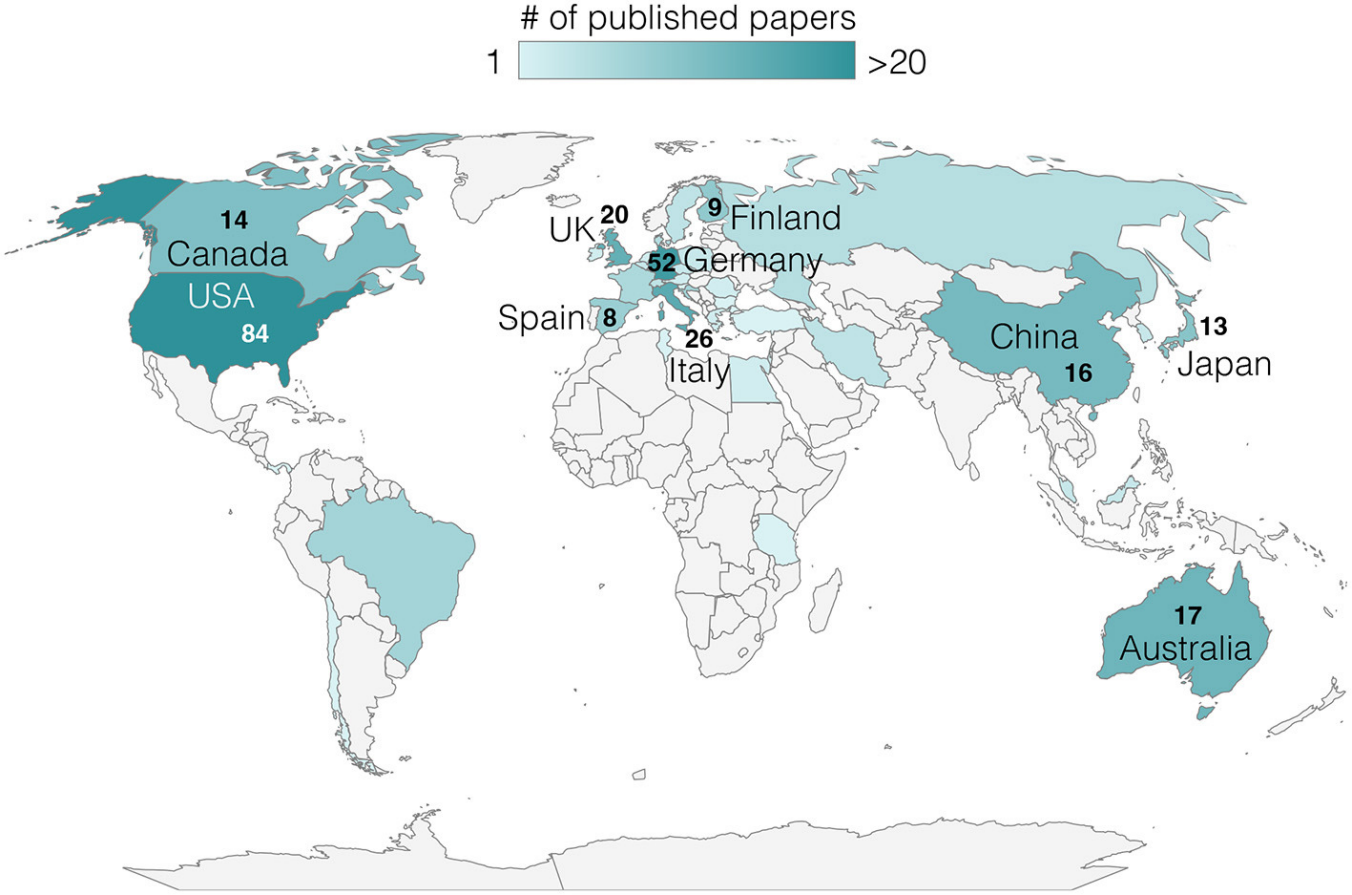
Map of published articles using NIBS to study speech motor aspects. Colored countries have contributed to one or more manuscripts, as indicated by the color bar above. The top 10 countries that most contributed to the field are highlighted, and the number of articles is indicated.

**Figure 2:**
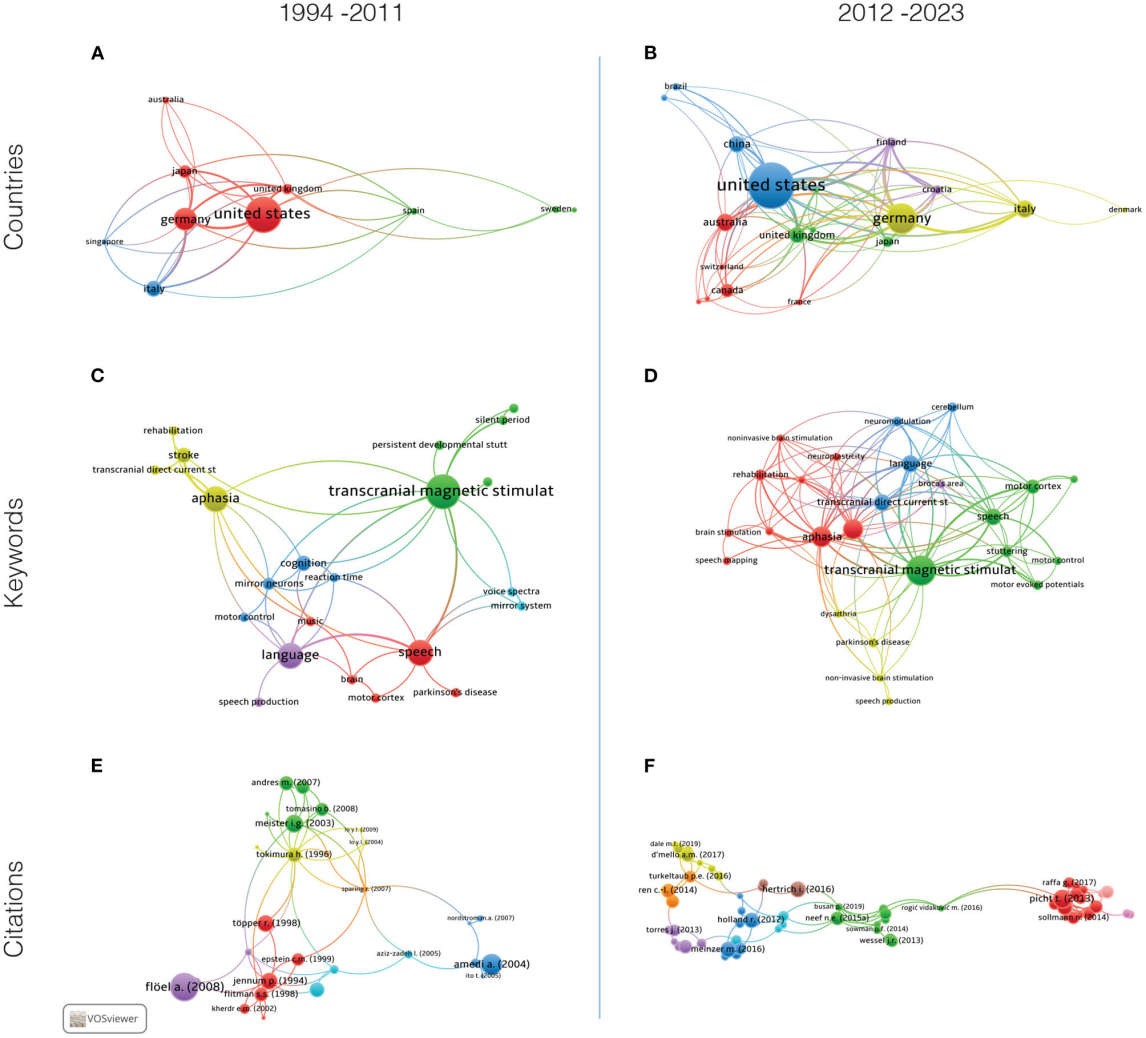
Visual displays of bibliographic data on the use of NIBS for speech motor aspects in two different time periods. Items analyzed were countries **(A, B)**, co-occurrence of keywords **(C, D)**, and citations **(E, F)**.

In Figure 1, the heatmap of all countries that have published articles that met the selection criteria is shown. The top 10 countries that contributed with most published articles to the field in the past 30 years are highlighted, with the total number of articles indicated. Geographical analysis in Figure 2A shows that from 1994 to 2011, nine countries were the sources of the most cited documents on NIBS and speech, while 17 countries were identified from 2012 to 2023 (Figure 2B), indicating the decentralization of this research field. The United States, Germany, the United Kingdom, and Australia are constant in their prominence across both time periods.

Keywords' co-occurrence grew from 153 to 469 over the 29 years analyzed, with 21 keywords divided into six clusters in 1994–2011 and 22 keywords divided into five clusters in 2012–2023 (Figures 2C, D, and Table 1), which demonstrates the diffusion of NIBS in various speech research subfields. Transcranial direct current stimulation and transcranial magnetic stimulation were constant across both time periods (Figures 2C, D).

Finally, document citation analysis identified seven citation clusters between 1994 and 2011 and seven different clusters between 2012 and 2023. Examples of the most cited studies in the first time period are the studies by Jennum et al. (1994) and Tokimura et al. (1996), whereas the second time period has studies by Picht et al. (2013); Busan et al. (2019), and Chesters et al. (2018) (Figures 2E, F). This section is further discussed below.

## Discussion

### The insertion of neuromodulation in speech-related fields

We have presented a comprehensive analysis of the scholarly intersection between speech and non-invasive neuromodulation through a bibliometric review of relevant literature. Keyword analysis shows that before 2012, TMS was the technique most frequently featured in the literature. The recurrence of some keywords associated with TMS illustrates the exploratory nature of early studies, attempting to modulate whole networks (“language”, “speech”) and possible relationships between speech and overall behavior and comprehension (“cognition” and “mirror neurons”). There are also researchers trying to bridge some of the theoretical knowledge with clinical treatment, as disorders such as aphasia, stuttering, and Parkinson's (which has an associated dysarthria aspect) appear in the keywords. As we move further into the 2010s, those applications are established and remain relevant in research. New keywords that appear at this time are “neuroplasticity”, probably due to continuous corroboration of the neuroplastic effects provided by repeated TMS and tDCS protocols; and “cerebellum”, probably derived from the still-growing explorations of cognitive and noncognitive functions of the cerebellum (Bostan and Strick, 2018; Lametti et al., 2018; Van Overwalle et al., 2020; Wilkinson et al., 2020).

Key studies from 1994 to 2012 reflect a strong initial focus on the mapping of language as a whole in humans, including speech functions. A few examples of such studies include Meister et al. (2003) and Flöel et al. (2008). All those studies use mainly TMS as an exploration tool or rehabilitation. The red cluster (Figure 2E) seems to represent some of the base work for further explorations, such as Tokimura et al. (1996), that first binds lateralized speech and motor excitability. We see the pioneer explorations of varied aspects of the language system by TMS (Flitman et al., 1998; Töpper et al., 1998; Epstein et al., 1999; Khedr et al., 2002). The other clusters, also composed of more recent studies, will be more driven toward the motor aspects of speech, with collaborations of the sensory system (Sparing et al., 2007; Mock et al., 2011), or possible parallels with jaw movements (Sowman et al., 2009), or possible singing processes (Lo and Fook-Chong, 2004).

From 2012 onward, there are emerging themes well defined by the clusters presented in Figure 2F. The red cluster, with Picht et al. (2013) as an evident representative, now focuses on non-invasive (mostly TMS) characterization of networks in healthy, awake humans, and on the neurosurgery context, symbolizing the shift toward clinical aspects we have commented above. Following these footsteps, the green and light blue clusters are mostly dedicated to stuttering (Chesters et al., 2018; Garnett et al., 2019; Yada et al., 2019), one of the speech disorders where motor aspects seem to be of most relevance. This builds upon earlier studies that first delved into stuttering, such as Sommer et al. (2003). The yellow cluster represents the exploration of cerebro-cerebellum networks related to speech motor control. Recent studies on how the cerebellum is a promising therapeutic target for neuromodulation probably played a part in this new avenue for modulating speech (Ferrucci et al., 2015, 2016; Grimaldi et al., 2016). The remaining clusters concern possible applications of neuromodulation for aphasia (Torres et al., 2013; Turkeltaub, 2015; Meinzer et al., 2016). The appearance of aphasia in motor explorations of language may seem odd, as aphasia is primarily not considered a speech motor disorder, but it seems to derive from the desire to explore the motor system as a gateway into recovery, or the possible unexplored motor aspects of aphasia.

### Current and future technique uses

Transcranial magnetic stimulation (TMS) was the pioneer technique in neuromodulation, created in 1985 (Barker et al., 1985), and it has been explored both in research (Thiel et al., 2006; Thielscher et al., 2015) and therapy (Lotze et al., 2006; Grossman et al., 2017). It was the main option for brain modulation until 2000 when Transcranial Direct Current Stimulation (tDCS) was created by Nitsche and Paulus. Since then, there is intense growth of research in this area, which might be due to the appearance of an option that is cheaper and easier to apply (TMS must always be applied by a trained physician due to safety issues). We also see in our data tDCS growing as another NIBS option (Torres et al., 2013; Chesters et al., 2018; Stahl et al., 2019).

From 1994 to 2011, there is a clear dominance of TMS for language, changing radically over the last 10 years when it starts to share more space with tDCS. “tDCS” as a keyword has 27 occurrences from 2012 to 2023, whereas, in the 1994–2011 period, it has only two occurrences (data not shown). For comparison, “TMS” has 23 appearances in the earlier period and 91 for the later period. Considering tDCS is presented as a tool in two seminal studies by Nitsche and Paulus in 2000 and 2001, this speaks to the time necessary for a technique to be inserted in any specific field. Its rapid growth may be a consequence of specific characteristics of tDCS in comparison to TMS, such as being a cheaper technique that demands less operational expertise (Priori et al., 2009).

There were no studies exploring transcranial random noise stimulation (tRNS) in our results. Only two of them explored transcranial alternating current stimulation (tACS); one about speech comprehension in people with aphasia post-stroke (Xie et al., 2022), and the other discussed proposed mechanisms for the effects of this modulation (Vogeti et al., 2022). This low number of studies might be due to a temporal bias as they have been developed more recently (Antal et al., 2008; Terney et al., 2008). There is, however, potential for these recent NIBS techniques in the field.

More recent NIBS techniques such as tRNS and tACS have been used for studying language but largely as markers of cognitive performance than as a process itself or mostly speech perception. Outside of our Scopus string focus, most of the studies using tACS or tRNS explore possible mechanisms of modulation and potential uses for cognition and motor rehabilitation in general. Few studies using tACS for language specifically focus on speech perception (Rufener et al., 2016a,b; Baltus et al., 2018; Riecke et al., 2018; Wilsch et al., 2018; Nooristani et al., 2021) and aphasia due to stroke (Fedorov et al., 2010). Similarly, for tRNS, there are studies found on general language, exploring the overall processing of auditory information (Rufener et al., 2017) and dichotic right-ear advantage (Prete et al., 2018). It may be evident that those themes, as well as the timing of their appearance in tACS and tRNS studies, resemble themes previously explored by TMS and tDCS studies. As such, we are of the opinion that this suggests an ongoing phase of acquiring information about applications and the intricate workings of those newly developed methods, which will subsequently be employed in addressing particular motor disorders.

tACS has been used to facilitate speech perception (Zoefel and Davis, 2017; Zoefel et al., 2018) using synchronized oscillations, which might also be gateways to facilitating fluency of speech (Busan et al., 2019). For example, tACS has a strong potential for modulating subcortical brain regions (Hess, 2013; Hashimoto and Karima, 2017), opening up plenty of opportunities to noninvasively explore the insula and other areas of interest for language. In the same way, tDCS has established itself as an alternative to studying speech detriment to TMS, and tACS might find its own niche in non-invasive deep brain stimulation. Even though the mechanisms and clinical applications of random noise stimulation are still under discussion (Brancucci et al., 2023), there is evidence of its capacity to modulate the motor cortex (Potok et al., 2022) and enhance motor sequence learning (Terney et al., 2008). It could also be interesting for considering inter-individual differences in brain activities, as tRNS may be able to amplify oscillations relevant to a particular task being executed. Overall, both these techniques represent a totally new approach, where we modulate brain oscillations instead of firing rates, which may allow for more anatomical and/or function specificity (Heimrath et al., 2016).

Finally, to the best of our knowledge, this is the first attempt to provide a bibliometric perspective on the use of neuromodulation to study the motor aspects of speech. Other analyses, such as systematic reviews, are useful to provide a comprehensive perspective of any field. However, as reviews favor the evidence level of quality, they may fail to grasp some other relevant aspects. The bibliometric analysis allows us to see “the evolutionary nuances of well-established fields” (Donthu et al., 2021), where we can observe geographical and thematic trends, and this is what we aimed for here. We can see how the growth of speech production as a research field is tightly linked to the development of technology. Furthermore, as evidenced by the exponential increase in results that we have observed, researchers would do well to consider the upcoming techniques of neuromodulation as they hold great promise for their methodological questions on speech.

## Conclusion

In summary, the bibliographic analysis conducted in this study revealed trends related to geography, technology, and themes over the past three decades in the field of NIBS applied to speech. There has been a transition from physiological descriptions of language and speech production toward the validation of its therapeutic applications. Looking ahead, future research should explore promising techniques such as tACS and tRNS, which offer significant potential for investigating speech. By providing a historical perspective, this study aims to offer guidance to prospective researchers in the field, aiding their methodological decisions and leading to more robust findings.

## Data availability statement

The raw data supporting the conclusions of this article will be made available by the authors, without undue reservation.

## Author contributions

FVC conceived the study. WM and TB were responsible for data collection and initial analysis. All authors participated in the data analysis, manuscript drafting, contributed to the article, and approved the submitted version.
